# Factors Associated with the Anxiety, Subjective Psychological Well-Being and Self-Esteem of Parents of Blind Children

**DOI:** 10.1371/journal.pone.0162294

**Published:** 2016-09-07

**Authors:** Juan Jesús Sola-Carmona, Remedios López-Liria, David Padilla-Góngora, María Teresa Daza, José Manuel Aguilar-Parra, María Ángeles Salido-Campos

**Affiliations:** 1 National Organization of Spanish Blind (ONCE), Almería, Spain; 2 Department of Nursing Science, Physiotherapy and Medicine, Faculty of Health Sciences, University of Almería, Almería, Spain; 3 Department of Psychology, Faculty of Psychology, University of Almería, Almería, Spain; 4 Complejo Hospitalario Torrecárdenas. Junta de Andalucía. Almería, Spain; Universita degli Studi di Perugia, ITALY

## Abstract

The objective was to examine the connection of the personal, social and family context, educational variables with the levels of anxiety, subjective psychological well-being and self-esteem in a sample of 61 parents of blind children. Results suggest that parents present less anxiety when they have only one child, possess a technical degree, receive remuneration for their work, their child’s visual impairment is not progressive, their knowledge about their child’s disability is appropriate, and their leisure and labour possibilities have not been affected. Their psychological well-being is higher when they are married in first nuptials and perceive that their health is good. Their well-being is negatively related to reduced leisure, and self-esteem is lower when labour possibilities have been affected. In order for these families to achieve a more pleasant life, with greater psychological well-being, lower anxiety and higher self-esteem, professionals should be aware of the aspects with a negative impact.

## Introduction

For many years, there has been a consensus that the birth of a child with a physical or intellectual disability constitutes a stressful experience involving a number of demands and significant challenges to the family, which could affect parents’ physical and psychological health [1–6].

In the case of visual impairment, some authors have suggested that the impact of the diagnosis of blindness received by some families can affect its members’ health, resulting in low self-esteem and high levels of anxiety, as well as in unhealthy behaviours and psychosocial problems due to the decrease of social and family relationships [7–11]. However, other authors have suggested that the importance of the burden and the wear and tear of caring for a disabled child in these families have been exaggerated. Thus, a recent study with Spanish parents of blind children suggests that the process of adaptation and psychological adjustment to the child’s disability may currently be changing [12]. Results of this study showed that the measures of anxiety and subjective psychological well-being of parents of blind children do not show any significant statistical differences when compared to the mean values of parents of typically developing children (e.g., families in a normalised population from these validated questionnaires), whereas their self-esteem measures were higher.

Despite these relevant developments, there is a lack of evidence that key processes such as acceptance play a role in parental adjustment. Within developmental family research, the concept of acceptance has traditionally been of interest. For example, MacDonald et al. (2010) used both the terms “acceptance” and “psychological adjustment” [13]. For these authors, acceptance is defined as the ability to take whatever is provided without trying to avoid experiences [13]. Checa et al. (2004) consider “psychological adjustment” to a disability as the person’s adaptation to the disease or disability and to its functional limitations [14, 15]. From a component-based conception of psychological adjustment, it is considered that the impact on some families of receiving the diagnosis of newborn child’s blindness may affect the following areas [8]: (a) psychological domain: increased anxiety, lower self-esteem, emotional maladjustment, passivity and loss of locus of control; (b) physical domain: mothers are more affected, as they are frequently devoted exclusively to caring for the child, leading them to neglect the rest of the family and to carry out high-risk behaviours for their health; (c) social domain: normally, due to feelings of shame or excessive responsibility, families tend to distance themselves from friends and to abandon social relationships; (d) family domain: relationships with the rest of the family become more difficult.

The literature and practical experience show that it is necessary to investigate variables that are related to these parents’ well-being and emotional serenity, aspects that are extremely important for the development of their disabled children. Intervention in these factors could turn the parents into positive agents of their children’s learning process, while providing professionals with very valuable information to guide orientations focused on the children and their families. The parent-professional relationship should lead to sharing the decision-making and planning processes, agreeing on common objectives [12,16–17].

The reviewed research shows that parents’ perception of and satisfaction with family-centred psycho-educational attention is very diverse and has been addressed from many different viewpoints. Therefore, more research is needed on the diverse personal or environmental variables that may be involved, modulating or influencing the anxiety and subjective psychological well-being of these families [6, 18–25]. Previous research has highlighted the importance of measuring psychological process variables, specifically with regard to parenting a child with a disability [26]. However, few research studies have explored the influence of multiple psychosocial variables on the anxiety, subjective psychological well-being and self-esteem of parents of blind children. Relevant aspects of the family setting and of the disability, if modifiable, would provide important information with which to plan psychosocial interventions with these families to achieve higher levels of health and quality of life. Evidence indicates that the family is in the best position to determine the needs and the well-being of the children [16].

The aim of this research was to investigate the relation between different personal variables, the socio-educational context of the family and the levels of anxiety, subjective psychological well-being and self-esteem of parents of visually impaired children. In this sense, previous literature concerning families of children with a disability leads us to hypothesise that these parents’ levels of anxiety will be higher when appropriate family support or a stable economic situation are lacking. However, both self-esteem and subjective psychological well-being may be affected by other variables more closely related to the level of satisfaction (e.g., the evolution of the visual impairment), the severity of the disability and the available support networks, for example, the psycho-educational attention received. Variables such as the parents’ knowledge of the disability, their expectations of improvement and their perception of their work and leisure possibilities could also be factors affecting their anxiety, well-being and self-esteem.

## Material and Methods

### Participants

The total population of the study comprised 95 parents from the province of Almería (southern Spain), whose children fulfilled the ophthalmic requirements for affiliation in National Organization of the Spanish Blind (O.N.C.E) and who were assisted by the specialized educational staff for blind and visually disabled people of this province (which has a total population of about 700.000 inhabitants). These parents were chosen as a function of their perfect knowledge of the Spanish language, efficient reading comprehension, and their continued contact with the orientation staff of the ONCE. Exclusion criteria for families were that their children were not receiving assistance from the educational orientation department (14 families) or their refusal to provide informed consent (20 refusals).

The final sample for this study consists of 61 parents, 45.9% (n = 28) men and 54.1% (n = 33) women, mean age 41.52 years (SD = 59). Of these fathers or mothers, 30% had only one child, 82% were married in first nuptials, 60.7% had no technical degree, and 60.7% received work wages.

The parents reported the following diagnoses for the children: 13.1% were totally blind, 34.4% were visually impaired, and 52% were visually impaired and also had intellectual and other physical disabilities. Of the children, 39.3% were diagnosed with congenital visual impairment, whereas 60.7% had acquired the disability. Regarding sex, 62.3% were boys and 37.7% were girls. The children’s mean age was 9.16 years (SD = 4.9).

### Procedure

This is a descriptive, cross-sectional study in which data were collected through questionnaires. First, the institutional review board of the Psychology Department in the University of Almería reviewed and authorized this project. The members of the orientation staff of the ONCE contacted the selected families by phone. After explaining the aims of the investigation to the parents, those who consented to participate in the study received the questionnaires with instructions by postal mail (each mother or father was requested to fill in the questionnaires and a consent form). The questionnaires were then returned to the researchers by postal mail in a stamped envelope provided with the questionnaires.

### Instruments

First, participants completed 21 questions that were elaborated ad hoc to collect information about their socio-demographic data and some variables related to the children’s degree of impairment, the support networks available to the families, and the parents’ expectations and perceptions of how their child’s disability affected their social, family and working life (Table 1). In previous studies, all these dimensions of disability have been considered as independent variables that can play an essential role in these families’ psychological adjustment. This list of variables was also reviewed by a panel of experts of seven professors of the Specific Attention Team for People with Blindness and Visual Impairment from the province of Almería (Spain) and by 14 experts in socio-educational attention to children with disabilities from the school for children with special needs, “Princesa Sofía”.

**Table 1:** Independent Variables in Parents of Children with Disabilities.

VARIABLE	CLASSIFICATION OR DESCRIPTION	AUTHORS (by year of publication)
Parents’ Sex	Male	King, King, Rosenbaum & Goffin, 1999 [27]; Dunst, 1999 [28]; Femenías & Sánchez, 2003 [9]; King et al., 2003 [29]; Dempsey & Dunst, 2004 [30]; Emerson & Hatton, 2008 [31]; Eisenhower et al., 2009 [32]; Benzies et al., 2009 [33]; Hatton et al., 2010 [3]; Martorell, Gutiérrez-Recacha, Irazábal, Marsa, & García, 2011 [34]; Piazza, Floyd, Mailick & Greenberg, 2014 [35].
	Female	
Parents’ Age	< 40 years	King et al., 1999 [27]; Dunst, 1999 [28]; Femenías & Sánchez, 2003 [9]; King et al., 2003 [29]; Dempsey & Dunst, 2004 [30]; Emerson & Hatton, 2008 [31]; Eisenhower et al., 2009 [32]; Benzies et al., 2009 [33]; Hatton et al., 2010 [3]; Martorell et al., 2011 [34]; Piazza et al., 2014 [35].
	> 40 years	
Civil Status	First nuptials	Holroyd et al., 1975 [36]; Kazak, 1987 [37]; Singer e Irvin, 1990 [38]; Guralnick, 1998 [39]; Dunst, 1999 [28]; Badía, 2002 [40]; Córdoba & Verdugo, 2003 [41]; Mayo, 2006 [42]; Benzies et al., 2009 [33]; Mayo, 2011 [11].
	Other	
Number of Children	1 child	Badía, 2002 [40]; Calvo & González, 2004 [8]; Navarro, 2004 [43]; Limiñana et al., 2007 [44]; Eisenhower et al., 2009 [32]; Benzies et al., 2009 [33]; Hatton et al., 2010 [3]; Martorell et al., 2011 [34]; Mayo, 2011 [11].
	> 1 child	
Parents’ Technical Qualification	Yes	Dunst, 1999 [28]; Navarro, 2004 [43]; Dempsey & Dunst, 2004 [30]; Emerson & Hatton, 2008 [31]; Papadopoulos et al., 2011 [45]; Piazza et al., 2014 [35].
	No	
Labour Remuneration	Yes	Friedrich & Friedrich, 1981 [46]; Byrne & Cunningham, 1985 [47]; Dunst et al., 1986 [48]; Guralnick, 1998 [39]; Dunst, 1999 [28]; Badía, 2002 [40]; Calvo & González, 2004 [8]; Dempsey & Dunst, 2004 [30]; Bumbalo et al., 2005 [49]; Mayo, 2006 [42]; Mayo et al., 2007 [50]; Emerson & Hatton, 2008 [31]; Mayo, 2011 [11]; Piazza et al., 2014 [35].
	No	
Child’s Sex	Male	Bingham & Smith, 2000 [51]; Córdoba et al., 2006 [52]; Piazza et al., 2014 [35].
	Female	
Child’s Age	< 7 years	Bingham & Smith, 2000 [51]; Dempsey & Dunst, 2004 [30]; Calvo & González, 2004 [8]; Córdoba et al., 2006 [52].
	7–12 years	
	> 12 years	
Child’s Visual Level	Total Blindness	Cummings, 1976 [53]; Holroyd & MacArthur, 1976 [54]; Wallander et al., 1988 [55]; Beckman, 1991 [56]; Krauss, 1993 [57]; King et al., 1999 [27]; Troster, 2001 [58]; Badía, 2002 [40]; King et al., 2003 [29]; Calvo & González, 2004 [8]; Mayo, 2011 [11].
	Visually Impaired	
	Multi-deficiency	
Age of Diagnosis	Congenital	King et al., 1999 [27]; King et al., 2003 [29]; Calvo & González, 2004 [8]; Mayo, 2011 [11].
	Acquired	
Visual Degeneration	Yes	Calvo & González, 2004 [8]; Mayo, 2006 [42].
	No	
Sleep Disorder	Yes	King et al., 1999 [27]; Badía, 2002 [40]; King et al., 2003 [29]; Calvo & González, 2004 [8]; Mayo et al., 2007 [50]; Mayo, 2011 [11].
	No	
Eating Habits	Appropriate	King et al., 1999 [27]; Badía, 2002 [40]; King et al., 2003 [29]; Calvo & González, 2004 [8]; Mayo et al., 2007 [50]; Mayo, 2011 [11].
	Inappropriate	
Development of the blind child	Less than expected	Mahoney & Bella, 1998 [23]; Dunst, 1999 [28]; O´Neil, Palisano, & Westcott, 2001 [59]; Calvo & González, 2004 [8]; Ponce, 2008 [60].
	Same as expected	
	Worse than expected	
Knowledge about the medical diagnosis	Appropriate	King et al., 1999 [27]; Lafuente, 2000 [61]; Calvo & González, 2004 [8]; Reich, Bickman, & Helflinger, 2004 [62].
	Inappropriate	
Satisfaction with the psycho-educational attention	Appropriate	King et al., 1999 [27]; Calvo & González, 2004 [8]; Navarro, 2004 [43]; Reich et al., 2004 [62]; Van Schie, Siebes, Ketelaar, & Vermeer, 2004 [63].
	Inappropriate	
Expectations of the child’s improvement	Yes	King et al., 1999 [27]; King et al., 2003 [29]; Serradas, 2003 [64]; Mayo et al., 2007 [50].
	No	
Having a disabled child affects the social network	Yes	Dunst et al., 1986 [48]; Guralnick, 1998 [39]; King et al., 1999 [27]; Rosa & Ruf, 2001 [65]; King et al., 2003 [29]; Calvo & González, 2004 [8]; Emerson & Hatton, 2008 [31]; Eisenhower et al., 2009 [32]; Hatton et al., 2010 [3]; Martorell et al., 2011 [34].
	No	
A disabled child affects leisure time	Yes	Guralnick, 1998 [39]; King et al., 1999 [27]; Rosa & Ruf, 2001 [65].
	No	
A disabled child affects job opportunities	Yes	Guralnick, 1998 [39]; Dunst, 1999 [28]; King et al., 1999 [27]; Rosa & Ruf, 2001 [65]; Leyser & Heinze, 2001 [66]; Bumbalo et al., 2005 [49]; Mayo, 2011 [11].
	No	
Parents’ health	“My health is always good”	King et al., 1999 [27]; Calvo & González, 2004 [8]; Mayo et al., 2007 [50]; Brehaut et al., 2009 [7]; Hutchinson, et al., 2009 [10]; Kuhlthau et al., 2010 [67]; Mayo, 2011 [11].
	“I feel good sometimes”	
	“My health is never good”	

After providing the above data, the parents completed three internationally recognised and validated questionnaires:

- The Spanish version of the State-Trait Anxiety Inventory (STAI) developed by Spielberger et al. (1970) and adapted in Spain by Spielberger et al. (1982) [68–69]. We employed the 20-item STAI-Trait Scale (STAI-T), with response options ranging from 0 (almost never) to 3 (almost always). Guillén-Riquelme et al. (2011) reviewed the current psychometric properties of the STAI in a total of 1036 adults. Cronbach’s alpha reliability was .94 for State Anxiety (similar results to the original data) [70].
- The "Escala de Bienestar Psicológico" (EBP; in English, the Scale of Psychological Well-being) [71] includes 30 items that measure happiness and well-being or positive affect, with response options ranging from 1 (strongly disagree) to 5 (strongly agree). Cronbach’s alpha reliability was .93, and the concurrent validity of this subscale and the Oxford Happiness Questionnaire (OHQ) was .89 [72].
- The Rosenberg Self-Esteem Scale (RSES) [73], which is a widely used scale in social science research, consisting of 10 items about a person’s general self-beliefs. In the present study, we employed the Spanish version of the RSES adapted by Baños et al. (2000). Martín-Albo et al. (2007) assessed the internal consistency of this scale with Cronbach’s alpha [74–75]. The values obtained in the first and the second administration were .85 and .88, respectively. The value of the test-retest correlation was .84.

### Statistical Analysis

The relation between the above-mentioned variables was analyzed by comparing the parents’ scores on the anxiety, self-esteem and psychological well-being questionnaires according to various levels of personal variables and socio-educational context (Table 1). For these analyses, we used Mann-Whitney U and Kruskal-Wallis K tests. The effect size was quantified to establish the degree of dependency between the variables (using fixed statistics for the non-parametric tests: r for the Mann-Whitney U and Cramer’s V for the Kruskal-Wallis). The analyses were performed with the SPSS statistical programme, version 20.0 (S1 Dataset).

## Results

As shown in Table 2, significant statistical differences were found in anxiety as a function of the number of children, having a technical degree, labour remuneration and worsening of the visual pathology. Parents with less knowledge about their child’s disability displayed higher levels of anxiety than parents with a good level of knowledge, and the difference was statistically significant. The same was true concerning the parents who stated that their leisure time had changed because of having a blind child. The parents who reported that their labour possibilities had been affected by having a disabled child also showed a higher anxiety level.

**Table 2:** Comparative analysis of the variables of the study in relationship with the State-Trait Anxiety Inventory (STAI) through non-parametric tests (Mann-Whitney U and Kruskal-Wallis K).

Independent or Moderating Variables		Dependent variable: State-Trait Anxiety					
		N	M	Z/df	U/K	p	r/v
Parents’ Sex	Male	28	27.20	-1.54	355.50	.123	.197
	Female	33	34.23				
Parents’ Age	<40	26	29.44	-0.59	414.50	.554	.075
	>40	35	32.16				
Civil Status	1^st^ nuptials	50	29.07	-1.81	178.50	.070	.231
	Other	11	39.77				
Number of children	1	18	23.25	-2.10	247.50	.035	.273
	+1	41	33.61				
Parents’ Technical Qualification	Yes	37	23.98	-2.49	275.50	.013	.318
	No	24	35.55				
Labour Remuneration	Yes	36	24.68	-2.98	222.50	.003	.388
	No	23	38.33				
Child’s Sex	Male	38	31.43	-0.25	420.50	.806	.032
	Female	23	30.23				
Child’s Age	<7	24	25.23	df 2	4.99	.082	.202
	7–12	17	31.91				
	>12	20	37.15				
Child’s Visual Level	Total Blindness	8	28.88	df 2	0.45	.798	.060
	Visually Impaired	21	29.62				
	Multi-deficiency	32	32.44				
Age of Diagnosis	Congenital	24	32.71	-0.61	403.00	.545	.078
	Acquired	37	29.89				
Visual Degeneration	Yes	13	41.62	-2.43	174.00	.015	.311
	No	48	28.13				
Child’s Sleep Disorders	Yes	18	35.11	-1.17	313.00	.241	.149
	No	43	29.28				
Eating Habits	Appropriate	21	23.98	-0.22	252.50	.825	.032
	Inappropriate	25	23.10				
Development of the blind child	Less than expected	40	31.86	df 2	0.82	.662	.081
	Same as expected	11	32.09				
	Worst than expected	10	26.35				
Knowledge about child’s disability	Appropriate	55	29.07	-2.57	59.00	.010	.329
	Inappropriate	6	48.67				
Satisfaction with psychoeducational attention	Appropriate	51	29.38	-1.61	172.50	.108	.206
	Inappropriate	10	39.25				
Expectations of child’s improvement	Yes	54	30.48	-0.63	161.00	.526	.080
	No	7	35.00				
Disabled child affects social network	Yes	26	35.73	-1.79	332.00	.073	.216
	No	35	27.49				
Disabled child affects leisure	Yes	38	35.75	-2.69	256.50	.007	.344
	No	23	23.15				
Disabled child affects job opportunities	Yes	30	35.20	-2.66	249.00	.008	.340
	No	28	23.39				
Parents’ Health	My health is always good	31	27.08	df 2	4.45	.108	.191
	I feel good sometimes	29	34.34				
	My health is never good	1	55.50				

Regarding psychological well-being (Table 3), statistically significant differences were found for the variables civil status and leisure: parents who reported that they were married in first nuptials or that their leisure time was not affected by having a blind child displayed higher/better subjective well-being than parents who stated that their leisure time had been affected. There was also a significant effect of the parents’ perception of their own health: parents who indicated that “their health was always good” showed a higher level of psychological well-being than parents who reported that they were “sometimes feeling good” or those who said they “never had good health”.

**Table 3:** Comparative analysis of the variables of the study in relationship to the Scale of Psychological Well-being through non-parametric tests (Mann-Whitney U and Kruskal-Wallis K).

Independent or Moderating Variables	Dependent variable: Psychological Well-being							
		N		M	Z/df	U/K	p	r/v
Parents’ Sex	Male		28	34.13	-1.26	374.50	.205	.161
	Female		33	28.35				
Parents’ Age	<40		26	29.40	-0.65	413.50	.545	.083
	>40		35	32.19				
Civil Status	1^st^ nuptials		50	33.38	-2.23	156.00	.026	.285
	Other		11	20.18				
Number of children	1		18	29.11	-0.40	353.00	.687	.050
	+1		41	31.10				
Parents’ Technical Qualification	Yes		37	32.65	-0.58	404.50	.560	.074
	No		24	29.93				
Labour Remuneration	Yes		36	33.07	-1.72	303.50	.086	.223
	No		23	25.20				
Child’s Sex	Male		38	30.34	-0.37	412.00	.710	.047
	Female		23	32.09				
Child’s Age	<7		24	31.00	df 2	0.411	.814	.058
	7–12		17	33.03				
	>12		20	29.28				
Child’s Visual Level	Total Blindness		8	28.25	df 2	2.307	.315	.137
	Visually Impaired		21	35.76				
	Multi-deficiency		32	28.56				
Age of Diagnosis	Congenital		24	26.81	-1.48	343.50	.138	.189
	Acquired		37	33.72				
Visual Degeneration	Yes		13	29.42	-0.36	291.50	.718	.046
	No		48	31.43				
Child’s Sleep Disorders	Yes		18	24.92	-1.73	277.50	.083	.221
	No		43	33.55				
Eating Habits	Appropriate		21	20.07	-1.58	190.50	.112	.233
	Inappropriate		25	26.38				
Development of the blind child	Better than expected		40	30.80	df 2	0.24	.887	.044
	Same as expected		11	33.14				
	Worst than expected		10	29.45				
Knowledge about child’s disability	Appropriate		55	31.78	-1.04	122.00	.298	.133
	Inappropriate		6	23.83				
Satisfaction with psycho-educational attention	Appropriate		51	32.62	-1.60	172.50	.108	.205
	Inappropriate		10	22.75				
Expectations of child’s improvement	Yes		54	31.69	-0.84	180.00	.417	.107
	No		7	25.71				
Disabled child affects social network	Yes		26	26.15	-1.84	329.00	.066	.235
	No		35	34.60				
Disabled child affects Leisure	Yes		38	26.74	-2.41	275.00	.016	.308
	No		23	38.04				
Disabled child affects job opportunities	Yes		30	25.97	-1.65	314.00	.099	.211
	No		28	33.29				
Parents’ Health	My health is always good		31	38.65	df 2	12.44	.002	.317
	I feel good sometimes		29	23.62				
	My health is never good		1	8.00				

Regarding self-esteem, we only found a significant effect for the variable related to parents’ perception of how having a disabled child affected their work possibilities (Table 4). Parents who reported that having a disabled child affected their work possibilities displayed lower self-esteem than those who stated that their work possibilities were not affected.

**Table 4:** Comparative analysis of the variables of the study in relationship to the Rosenberg Self-Esteem Scale through non-parametric tests (Mann-Whitney U and Kruskal-Wallis K).

Independent or Moderating Variables	Dependent variable: Self-esteem scale							
		N		M	Z/df	U/K	p	r/v
Parents’ Sex	Male		28	30.63	-0.15	451.50	.879	.019
	Female		33	31.32				
Parents’ Age	<40		26	31.94	-0.35	430.50	.720	.044
	>40		35	30.30				
Civil Status	1^st^ nuptials		50	31.67	-0.63	241.50	.528	.080
	Other		11	27.95				
Number of children	1		18	28.28	-0.64	338.00	.517	.083
	+1		41	31.45				
Parents’ Technical Qualification	Yes		37	33.96	-1.05	373.00	.293	.134
	No		24	29.08				
Labour Remuneration	Yes		36	31.57	-0.88	357.50	.378	.114
	No		23	27.54				
Child’s Gender	Male		38	29.00	-1.13	361.00	.257	.144
	Female		23	34.30				
Child’s Age	<7		24	32.75	df 2	4.129	.127	.183
	7–12		17	35.97				
	>12		20	24.68				
Child’s Visual Level	Total Blindness		8	32.81	df 2	0.205	.903	.040
	Visually Impaired		21	31.71				
	Multi-deficiency		32	30.08				
Age of diagnosis	Congenital		24	29.88	-0.40	417.00	.689	.051
	Acquired		37	31.73				
Visual Degeneration	Yes		13	26.62	-1.01	255.00	.314	.129
	No		48	32.19				
Child’s Sleep Disorders	Yes		18	25.81	-1.48	293.50	.138	.189
	No		43	33.17				
Eating habits	Appropriate		21	23.93	-0.19	253.50	.842	.028
	Inappropriate		25	23.14				
Development of the blind child	Better than expected		40	30.94	df 2	0.543	.762	.066
	Same as expected		11	28.41				
	Worst than expected		10	34.10				
Knowledge about child’s disability	Appropriate		55	32.20	-1.74	114.50	.083	.223
	Inappropriate		6	19.08				
Satisfaction with psycho-educational attention	Appropriate		51	31.88	-0.88	210.00	.379	.113
	Inappropriate		10	26.50				
Expectations of child’s improvement	Yes		54	31.34	-0.42	170.50	.682	.053
	No		7	28.36				
Disabled child affects social network	Yes		26	28.87	-0.81	399.50	.417	.103
	No		35	32.59				
Disabled child affects leisure	Yes		38	28.47	-1.43	341.00	.152	.183
	No		23	35.17				
Disabled child affects job opportunities	Yes		30	23.88	-2.63	251.50	.009	.337
	No		28	35.52				
Parents’ Health	My health is always good		31	34.53	df 2	2.511	.285	.142
	I feel good sometimes		29	27.36				
	My health is never good		1	27				

## Discussion

Our results suggest that some individual/personal, social and educational variables are associated with the level of anxiety, subjective psychological well-being and self-esteem of parents of blind children.

With respect to civil status, parents in first nuptials showed a higher level of well-being than parents who are remarried, single or divorced. This result is congruent with previous studies which have shown that couple support in families with disabled children acts as a protector against stress because effective family support helps families to perform their care functions with less psychological distress and more satisfaction [36, 40, 42]. Thus, when the marriage is solid and support is mutual, couples can better deal with problematic situations, which, according to Mayo (2011), serve to unite the family as a form of adaptation and even to improve the relationship [11, 41].

Regarding the number of children, in our study, approximately one third of the parents had only one child and they showed less anxiety than the parents who had more than one child. In this sense, authors like Navarro (2004) and Mayo (2011) have suggested that the parents of children with disability have doubts about having more children because these children could also present the disability or pose a new burden [11, 43].

With regard to educational and economic level, parents who had a technical degree or who received labour remuneration showed less anxiety than those who were unemployed, indicating that these factors can act as buffers against anxiety in difficult situations. Previous studies have also reported a relation between stress and families’ low socio-economic level [42, 46, 48–50]. However, other authors like Byrne et al. (1985) or Badía (2002) have not found a clear relationship [40, 47]. They justify this because, in Spain, people generally have access to social, health and educational services, regardless of their socio-economic level. Even so, in Spain, where social services are generalised, parents do not always consider them sufficient to meet their children’s needs, so they must seek complementary private resources [76–77].

Concerning the disability itself, there is a great variety of opinions about the impact of the severity of the children´s disability on the parents’ health status. Authors like Beckman (1991), Cummings (1976) and Holroyd et al. (1976) found that the severity of the child’s disability affects the parents’ stress [53, 54, 56]. However, authors like Krauss (1993) and Wallander et al. (1988) did not find any relationship between the level of stress and the level of disability [55, 57]. Regarding visual impairment, Troster (2001) found no significant differences in the stress of mothers of visually impaired children and mothers of completely blind children [58]. Upon comparing the current study with that of Troster (2001), there are no significant differences in parental stress between parents of visually impaired, totally blind, or multi-deficient children [58]. This could be because visually impaired children may reach very high levels of autonomy when their socio-familiar environment is appropriate, and use of the major advances in optics, technology, and computer science is promoted. However, the child’s progressive visual impairment had a significant impact on the parents’ anxiety in our sample of parents, indicating that uncertainty about the development of the impairment and fear of the total loss of their children’s vision may produce high levels of anxiety in families. In this sense, Mayo (2006) also indicated that, in cases of visual degeneration, the family will suffer higher levels of tension [42].

The results of this study do not show that the moment of blindness diagnosis is related to the dependent variables of parental anxiety, subjective psychological well-being, or self-esteem. However, they could indicate that, when parents have adequate information about their child’s disability, their anxiety is lower than when they do not. Knowing the principal features of the child´s disability can help parents to feel more secure and self-confident about educating and working with a blind child. If the knowledge is inappropriate, this could lead to attitudes of shame and overprotection, making normalised development more difficult. Therefore, correct knowledge about the visual impairment should lead to better health and lower anxiety.

With regard to the parents’ reporting that their child’s development had been “better, the same or worse than expected”, no statistically significant differences were found in anxiety, subjective well-being or perceived self-esteem. However, subjective psychological well-being was associated with these variables when the parents thought they had good health. This corroborates that subjective psychological well-being can be used as an indicator of health, in agreement with Fierro’s (2006) model of bipolar health [78].

On another hand, parents who thought that their labour possibilities had been affected presented a higher level of anxiety and lower self-esteem. It seems logical that a child with this disability will need more care and dedication, and this can make the parents—especially the mothers—reconsider their job possibilities. Rosa et al. (2001) revealed the loneliness and reduced social contact of these families, finding that the mothers also have fewer opportunities to join the labour market [65]. Mayo (2011) also agreed that having a visually impaired child can cause tension in working relationships, and Calvo et al. (2004) found that parents of disabled children may feel they are being attacked by society [8, 11].

As future areas of research related to this work, we suggest intervention research to promote better parenting or family functioning for families with a disabled child. For example, it is important for these families to cultivate their social relations. With suitable counselling and organisation of their free time, family members can learn to deal with the potential discomfort caused by people’s stares and indiscreet questions about the blind child, the awkwardness of introducing him or her to society. Professionals should also participate directly in other life aspects that could lead to better psychological well-being and lower anxiety, such as counselling the parents and motivating them to seek appropriate knowledge about their child´s visual impairment, to care for their social network, and to enjoy their leisure time.

Among the limitations of this investigation, we note the reduced sample size, as not all the parents contacted consented to participate in the study, and this sample was drawn from a group of parents whose children were assisted by the specialized educational staff of the ONCE. The responses to the items by parents who are not in continued contact with the orientation staff can be expected to be very different from responses of those who are. With regard to the selected sample, not all the blind children had the same degree of disability, and it may also be useful to compare parents of blind children with a control group of parents without a child with a disability. Therefore the generalizability of the results of this study is limited and should be considered with this caveat in mind.

In spite of these limitations, the results of this study could contribute to promote scientific evidence-based studies to improve the outcomes of interventions in families with disabled children.

Summing up, this study suggests that the parents of blind children show less anxiety when they have only one child, they possess a technical degree, have a remunerated job, their child´s visual impairment is stabilised, they consider that their knowledge of their child’s disability is appropriate, and their leisure and labour possibilities have not been affected. They have a greater subjective psychological well-being if they are married in first nuptials and they think their health is good. Their self-esteem improves when they perceive that their job opportunities are not affected because of having a blind child.

The practical implications of this research should influence interventions with these families. In order for these families to achieve a more pleasant life, with greater psychological well-being, lower anxiety and higher self-esteem, professionals should be aware of the aspects with a negative impact.

## Supporting Information

S1 Dataset MatrizONCEenglish (SAV)
